# Modelling Analysis on Dietary Patterns and Oral Health Status among Adolescents

**DOI:** 10.3390/ijerph192215255

**Published:** 2022-11-18

**Authors:** Huay Woon You

**Affiliations:** Pusat GENIUS@Pintar Negara, Universiti Kebangsaan Malaysia, Bangi 43600, Selangor, Malaysia; hwyou@ukm.edu.my

**Keywords:** dietary patterns, adolescents, oral health, modelling analysis

## Abstract

Adolescence is a crucial stage between childhood and adulthood during which an individual learns new behaviours and practices including dietary patterns. This study aimed to examine the diet and oral health status among adolescents, and employed a structured questionnaire with three sections, namely, demographic, Adolescents’ Food Habits Checklist (AFHC) and the Kayser–Jones Brief Oral Health Status Examination (BOHSE). The AFHC was formulated consisting of 23 items to collect information about dietary patterns with respect to food purchase, preparation and consumption habits. Meanwhile, the BOHSE contained nine items to evaluate the oral conditions of adolescents. The relationship between dietary pattern and oral health in adolescents was investigated. A total of 160 adolescents were randomly selected in this study. The data analysis was presented in the form of tables. This study adhered to the STROBE checklist’s Guidelines for Systematic Reporting of Examination. According to the findings, food consumption dietary patterns among adolescents had the highest mean score (4.475). This demonstrates that adolescents practiced healthy food consumption. A significant positive correlation was found between food purchase, food preparation, food consumption and dietary patterns. Moreover, females had a slightly higher mean score than males, showing that females have a healthier diet status than males. This study can serve as points of reflection and recommendations on dietary patterns and oral health status.

## 1. Introduction

Diet is defined as the type of food we consume daily and continuously [1]. Dietary patterns reflect the way to practice a diet, including food purchase, preparation and consumption. A balanced diet provides essential nutrients for optimum overall oral and general health [2]. Diet is often the first to be reviewed regarding changes in our health conditions, and in particular, oral health. Diet plays a major role in promoting and maintaining good health and preventing diseases. Consistent nutrition guidelines are essential to improving health.

The variety of food consumed has respective effects. According to the American Dental Association [3], calcium-rich foods such as low-fat or fat-free milk, yogurt and cheese, fortified soy drinks and tofu, canned salmon, almonds and dark green leafy vegetables help promote strong teeth and bones. Moreover, vegetables such as peppers, broccoli, potatoes and spinach are rich in vitamin C, which is important to maintain healthy gums. Foods containing casein such as cheese or milk can also assist in the prevention of dental decay. Eggs, fish, lean meat dairy, nuts and beans contain phosphorus, which is good for strong teeth. Both calcium and phosphorus play a critical role in dental health by protecting and rebuilding tooth enamel. Fruits and vegetables are a good choice for healthy teeth, since they have abundant water and fibre. They also help in stimulating saliva production, which washes harmful acids and food particles away from teeth and helps to neutralise acid to protect the teeth from decay.

Based on The Sun [4], nine out of ten Malaysian adults have experienced periodontal disease and dental caries. Based on the study by the Health Ministry, 40 to 75% of school children have dental caries, and 76% of five-year-olds have experienced caries. Across the world, it is stated that 60–90% of school children and nearly all adults have dental cavities [5]. One of the main factors that affect oral cavities is diet [6]. To ensure good oral health, it is recommended that one has an appropriate and adequate nutritious diet. Caries on the teeth can be brought on by poor dietary decisions. Dental caries indicate improper food choices and irregular eating habits. The frequency of consumption of foods high in carbohydrates is the most crucial element in the association between diet and oral health. According to Morikava, et al. [7], there is a karyogenic effect of food intake with dental caries. This can be seen in the intake of carbohydrates, especially sucrose, which affects one’s tooth strength.

Blackwell and Dalton [8] investigated oral health and concluded that it is important for our body’s overall performance and health. They have stated that diseases such as obesity, cardiovascular diseases and dental caries all share the same type of risk factors such as carbonated drinks, fast food and sugary foods. Poor oral health during childhood will result in dental caries such as tooth decay and plaque, which can remain susceptible to development throughout the children’s lives [9]. Gil-Montoya, et al. [10] proposed that as part of general health, oral health is influential on multiple features of life, including self-esteem, interaction skills and work performance. Furthermore, oral health also contributes to one’s appearance as a whole. It is also discussed in the study that one of the main challenges of oral health is dental caries. These dental caries are often the result of a poor diet, with some additional factors such as aging and the exposure of our oral cavity to unfavourable surroundings such as high acidity.

According to the World Health Organisation [11], the concept of oral health is associated with quality of life. In this case, a healthy oral status is the result of careful care. In addition, mouth bacteria called Streptococcus will break down and digest simple sugars to produce acids that will dry out the surface of the tongue and mouth. The constant presence of sugary foods in the mouth will create an oral environment that has a low pH. The acid will dissolve minerals from the tooth surface, and the tooth develops caries. According to MyHEALTH Kementerian Kesihatan Malaysia [12], excessive sugar intake results in dental caries. In addition, as the frequency of sugar intake and the stickiness of sweet foods increase, the rate of caries’ formation will also increase. The function of self-cleansing with saliva is less effective if the food attached to the tooth surface is sweet and sticky. This shows that dietary patterns play an important role in oral health status.

Furthermore, improper dietary choices can cause dental caries. Accordingly, the lack of promotion of oral health has increased the negative oral health behaviours of the community. Adolescents are the community’s future leaders, and their views toward dietary patterns reveal how well they grasp the importance of oral health status. Meanwhile, the effects of good nutrition and oral health care will benefit overall health. Therefore, the effectiveness of dietary patterns on oral health is required among adolescents. The appropriate time to start cultivating lifelong healthy habits is during adolescence. Recent years have seen an increase in nutritional health and oral health issues, which have become a significant health problem. Evidence is needed to draw attention to the dietary patterns and oral health status of adolescents and to promote diet education and quality. National studies on dietary patterns and oral health status are limited compared with the existing international ones. For this reason, the current study aimed to examine the relationship between dietary patterns and oral health status of adolescents. This work is organised as follows. The research methodology that was employed in this study is discussed in the next section. Section 3 elaborates on the results, and a discussion of these results is presented in Section 4. Finally, the key findings are highlighted in the last section (Section 5).

## 2. Materials and Methods

The aim of this study was to investigate the dietary patterns and oral health status among adolescents from the lens of a modelling analysis. After the institution approval processes were completed, the researcher started to conduct the study. This research was a cross-sectional study. The Adolescents’ Food Habits Checklist (AFHC) and Kayser–Jones Brief Oral Health Status Examination (BOHSE) were employed to collect information from adolescents regarding their dietary patterns and oral health status, respectively. The AFHC and BOHSE were adopted from Johnson et al. [13], and Taub [14], respectively. The questionnaire adopted in this study contains 23 rating scale items from AFHC, 9 items with a three-point Likert scale from BOHSE and a section at the beginning of the questionnaire was used to collect demographic information from the adolescents. The BOHSE was the tool that could be used via observation of the oral cavity and surrounding tissues, which covered oral health status. The terms in the questionnaire have been slightly modified according to the respondent’s background. The modification was as follows: the “severely fissured” has been replaced with “severely cracks” in the questionnaire. The researcher will clarify to the adolescents if there is any confusion. Meanwhile, the questionnaire was in the English language.

**Figure 1:**
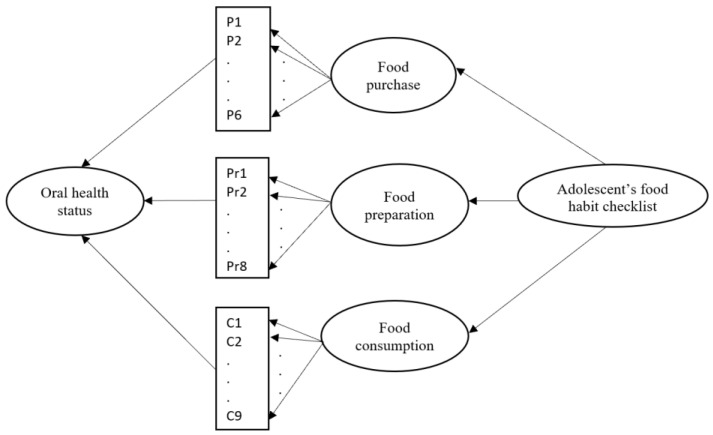
Research model.

Through the lens of the AFHC questionnaire, the items included three key dimensions, which were food purchase, food preparation and food consumption. From the dietary patterns, the adolescents’ oral health statuses were examined. The research model is shown in Figure 1. From the figure, there were six items, eight items and nine items that addressed the food purchase, food preparation and food consumption, respectively. All adolescents were informed about the study, and those who participated were required to provide an informed consent. The participation of the adolescents was entirely voluntary, and they had the right to decline to participate without any consequences, at any time. Moreover, the participation of the respondents was anonymous.

**Figure 2:**
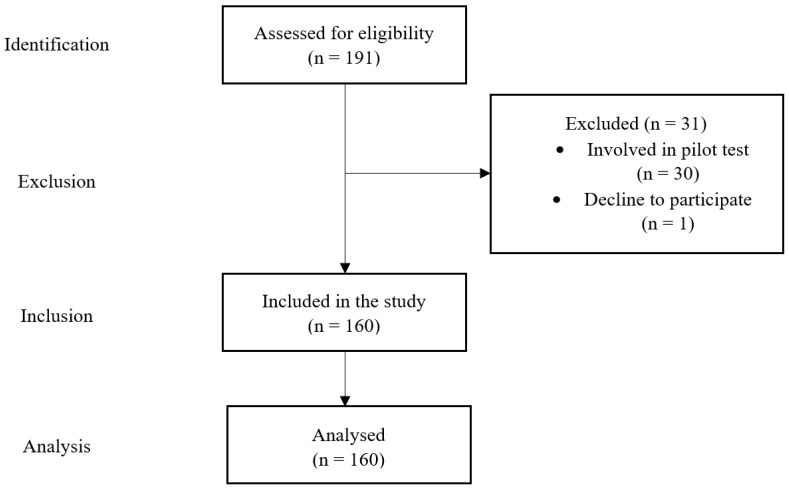
Flowchart describing population and sample.

This study utilised the paper survey. Hence, this provided an opportunity to the adolescents if they had any questions on the survey. The study location was at a pre-university centre. The university’s name was kept anonymous. The adolescents were pre-university students. Simple random sampling was employed to recruit adolescents in this study. The population size was 191 adolescents, and the participants were 160 adolescents, of which, 65 were boys, and 95 were girls. Based on the [15,16], for the population size 191, the corresponding sample size needed was 127. A total of 161 questionnaires were distributed. The response rate in this study was 99.38%. Figure 2 shows the flowchart describing the population and sample. A pilot test was conducted among 30 adolescents through a simple random sampling method. A reliability test was conducted on the collected data using IBM SPSS Statistics Version 25. The Cronbach’s Alpha values for the AFHC and BOHSE were 0.85 and 0.80, respectively. This indicated that all items were in a good level of reliability.

When answering the questionnaire, the respondents were aware of their dietary patterns and oral health status. For the oral health examination with the BOHSE tool, two final year students in dentistry have been recruited and they are refused to be named. For this, training and calibration among the examiners have been conducted before the data collection. In addition, the responses were confirmed with the adolescents. All the collected data were analysed using IBM SPSS Statistics Version 25. Descriptive statistics and inferential statistics were included in the data analysis. The results were presented in the form of tables. Descriptive statistics, for example, mean, standard deviation, frequency and percentage were used to describe the collected data. For inferential statistics, the correlation coefficient was used to examine the relationship between the variables. Meanwhile, a hypothesis *t* test and multivariate analysis of variance (MANOVA) were used to investigate the hypothesis of if there is a difference between the gender on the dependent measures. Here, five dependent measures were examined, namely, overall dietary patterns, food purchase, food preparation, food consumption and oral health status. Meanwhile, multiple regression was used to identify the variability in oral health status that can be accounted for by food purchase, food preparation and food consumption.

## 3. Results

Among the adolescents, the percentage of females (59.4%) was higher than that of males (40.6%) (see Table 1). For the AFHC questionnaire, one point was given for each healthy dietary pattern and no point was given for any unhealthy dietary pattern. In view of this, the high mean score indicated healthy dietary patterns among adolescents. On the contrary, a 3-point Likert scale was used for the items in the BOHSE, which were 0, 1 and 2 points. Here, a higher mean score indicated an unhealthy oral status.

Table 1 demonstrates the mean and standard deviation for food purchase, food preparation, food consumption, dietary patterns and oral health status. In addition, the gender of the adolescents is included in Table 1. The mean and standard deviation for the nine items from the BOHSE have been displayed in Table 1. From the table, the food consumption dietary patterns among adolescents had the highest mean score (4.475), followed by food preparation (4.2375). The lowest mean score was food purchase (2.3875). This demonstrates that adolescents practiced healthy food consumption in general. Meanwhile, the means of dietary patterns and oral health status were 11.1000 and 1.7125, respectively.

Among the nine items in the BOHSE, the item “lips” had the highest mean (standard deviation) of 0.5063 (0.53784). This indicates that the majority of the adolescents had unhealthy lips in comparison with other items in the BOHSE. This was followed with the items “condition of natural teeth” and “oral cleanliness”, with 0.3125 and 0.2188, respectively. These two items also required attention among adolescents to maintain good oral health. On the contrary, the lowest mean (standard deviation) score was the item “condition of artificial teeth (if present)”, with 0.0438 (0.25934). This demonstrates that there were adolescents with artificial teeth. This portrayed an important phenomenon to examine in oral health, regardless of the age.

To examine the relationship between the variables, the correlation coefficients between the variables were calculated and presented in Table 2. Significance presented in the table is indicated where * represents a *p*-value = 0.000. The bivariate correlation between food purchase, food preparation, food consumption and dietary patterns was positive and significant, with a *p*-value = 0.000. This shows that there was sufficient evidence to suggest that a positive correlation existed in the population. In contrast, there was a negative relationship between oral health status and food purchase, food preparation, food consumption and dietary patterns. In addition, the negative relationship between these measures was non-significant.

Table 3 displays the mean and significance of gender differences for the five measures. Data were analysed independently for boys and girls. A non-significant difference emerged for all the variables. This reports that there was no difference between girls and boys in dietary patterns and oral health status. To have a clear comparison, a multivariate analysis of variance (MANOVA) was used to examine the effects of gender on overall dietary patterns and oral health status. The MANOVA was statistically non-significant, F(4, 155) = 0.538, *p*-value = 0.708, partial $\eta^{2}$ = 0.014, indicating the absence of any meaningful gender differences on overall dietary patterns and oral health status. This can also be observed from the means for each dependent variable, which is shown in Table 3.

To clarify the dimension of the AFHC that had the most significant impact on the oral health, multiple regression has been conducted in this study. In combination, food purchase, food preparation and food consumption accounted for a non-significant 1.4% of the variability in oral health status, R^2^ = 0.014, adjusted R^2^ = −0.005, F(3, 156) = 0.737, *p* = 0.532. Unstandardised (B) and standardised (β) regression coefficients and squared semi-partial (or “part”) correlations (sr^2^) for each predictor in the regression model are reported in Table 4.

## 4. Discussion

In this study, among food purchase, food preparation and food consumption, the highest mean score was obtained by food consumption. This shows that the adolescents practice healthy food consumption. A study by Ziegler et al. [17] showed that adolescents prefer the closest and fastest items in the selection of food. This is related to the environmental factors. This reflects that the patterns related to the autonomous selection of food need to be guided and practiced at a young age. According to Hormenu [18], there was less consumption of fruits and vegetables among adolescents. This finding is different in this study, where the mean consumption of fruits and vegetables among adolescents was 2.7875. This indicates that the adolescents in this study practiced the consumption of fruits and vegetables in their daily lifestyle. Apart from healthy food consumption, a healthy lifestyle, such as engaging in physical activities, is also essential [19].

The top three items with the highest mean reported in this study were “lips condition”, “condition of natural teeth” and “oral cleanliness”. The higher the mean score indicates the high agreements on the items with the unhealthy condition. These findings are similar to the results from [20], where the percent agreements were found on the items lips, natural teeth and oral cleanliness with 60.7%, 60.7% and 32.1%, respectively. The items lips, condition of natural teeth and oral cleanliness are closely related to each other. This is because the condition of natural teeth depends on oral cleanliness. Meanwhile, lips are the first defence on the oral health status. Saliva plays an important role in the lips, condition of natural teeth and oral cleanliness. The dietary pattern will influence the stimulation of saliva production. Saliva is essential in neutralising acids in protecting teeth decay. The item condition of artificial teeth (if present) had the most healthy condition with the lowest mean score of 0.0438. This indicates that adolescents do not have or have less broken teeth or missing teeth.

There is a negative correlation between oral health status and food purchase, food preparation, food consumption and dietary patterns. It is known that the higher the AFHC score, the healthier the dietary pattern. On the other hand, the higher the BOHSE score, the unhealthier the oral health status. The negative correlation shows that the higher the oral health score (unhealthy status), the lower the dietary score (i.e., unhealthy dietary patterns). These results are supported by Kiesswetter et al. [21], who stated that oral health characteristics are related to dietary patterns. Meanwhile, according to Alpert [22], oral health is influenced by many factors, including diet. Yamgai et al. [23] also found that there is a negative correlation between dental care and food choice. This demonstrates that food choices and eating behaviours play an important role. Adolescents’ level of nutrition knowledge occupies an essential position among individuals. The ability to obtain and comprehend nutritional knowledge along with the ability to make appropriate nutritional decisions are required at a young age.

It was also identified that there is no significant difference between gender with respect to food purchase, food preparation, food consumption, dietary patterns and oral health status. Bloom, et al. [24] conducted a qualitative study in identifying the diet quality and found that there is no significance difference between gender with respect to food preparation, food consumption and dietary patterns. According to Riad et al. [25], the gender-based differences are not statistically significant with respect to oral health behaviour. In view of this, there is no difference in gender in influencing food purchase, food preparation, food consumption, dietary patterns and oral health status. This highlights that the study participants have their own dietary patterns and oral health status that are influenced by the environment and background. Factors such as education can be considered in future studies.

The unstandardised regression coefficient for food purchase is −0.083. This means that after controlling food preparation and food consumption, a 1-unit decrease in food purchase will result in a predicted 0.083-unit decrease in oral health. Meanwhile, the standardised regression coefficient for food purchase is −0.072. In other words, after controlling food preparation and food consumption, a 1 standard deviation decrease in food purchase will result in a 0.072 decrease in oral health. This indicates that the purchase of unhealthy food will decrease the oral health status. This is similar to the results obtained from [26], which demonstrated that food choice motivates dietary patterns and oral health status. From the findings obtained, factors such as education and quality of life can be considered in future studies. This is because dietary patterns and oral health status have the potential to be influenced by the education background of the caregivers and the quality of life. This study provides new insights that dietary patterns and oral health status can been studied from various perspectives. Meanwhile, dietary patterns and oral health status among adolescents are worth focusing on, as adolescents will become adults and their behaviour will influence the next generation.

It should be emphasised that the number and distribution of the responses were the main limitations of this study. As a result, it shows that the findings are more reflective of the dietary patterns and oral health status of adolescents in this particular group, and they are not representative of the entire population of adolescents. In addition, scarcity of time caused this study to focus solely on a particular pre-university centre and age group. Upcoming studies may include different age groups in the study to have an overall understanding of the dietary patterns and oral health status.

## 5. Conclusions

This study examined the dietary patterns and oral health status among adolescents. In general, it is known that the dietary patterns affect oral health status. The dietary patterns and oral health status of adolescents were good. It was shown that the adolescents’ dietary pattern had an impact on their oral health status. For dietary patterns, a higher score indicated a healthier diet, while for oral health status, a lower score indicated a healthier status. It was discovered that gender had no influence on adolescents’ dietary patterns and oral health status. According to these findings, the dietary patterns and oral health status of individuals and societies should be established and raised to ensure a healthy diet and oral health status. In order to educate adolescents and families about dietary patterns and oral health status, ongoing interventional studies coordinated by education programmes are crucial for community health. In addition, school canteens need to sell nutritious foods. From an early age, adolescents should be guided to select the healthy food. Meanwhile, physical activity should be promoted and transformed into a lifestyle. Adolescents’ awareness should be raised, and appropriate health policies should be developed in regard to this issue, by creating education content. Future studies can consider employing different methods of data collection such as interviews for better triangulation of the results obtained.

## Figures and Tables

**Table 1:** Summary measures for demographic, food purchase, food preparation, food consumption, dietary patterns and oral health status.

	*n* (%)	Mean	Standard Deviation
Gender			
Male	65 (40.6%)		
Female	95 (59.4%)		
Food purchase		2.3875	1.50466
Food preparation		4.2375	1.94091
Food consumption		4.4750	1.97771
Dietary patterns		11.1000	4.51580
Oral health status		1.7125	1.73164
9 items from BOHSE			
Lips		0.5063	0.53784
Tongue		0.1063	0.30912
Tissue inside cheek, floor and roof of mouth		0.0938	0.33264
Gums between teeth		0.1813	0.41772
Saliva (effect on tissue)		0.0750	0.26422
Condition of natural teeth		0.3125	0.47830
Condition of artificial teeth (if present)		0.0438	0.25934
Pairs of teeth in chewing position		0.1750	0.41284
Oral cleanliness		0.2188	0.44399

**Table 2:** Correlation matrix between food purchase, food preparation, food consumption, dietary patterns and oral health status.

	1	2	3	4	5
1. Food purchase		0.558 *	0.485 *	0.786 *	−0.107
2. Food preparation	0.558 *		0.560 *	0.861 *	−0.088
3. Food consumption	0.485 *	0.560 *		0.840 *	−0.094
4. Dietary patterns	0.786 *	0.861 *	0.840 *		−0.115
5. Oral health status	−0.107	−0.088	−0.094	−0.115	

Note: * *p*-value = 0.000.

**Table 3:** Analysis for each measure by gender.

	Girls (*n* = 95)	Boys (*n* = 65)	Total (*n* = 160)	Significance of Gender Differences
Food purchase	2.4947	2.2308	2.3875	*t*(158) = −1.091, 0.277 (NS)
Food preparation	4.0000	4.0000	4.2375	*t*(158) = −1.283, 0.201 (NS)
Food consumption	4.5368	4.3846	4.4750	*t*(158) = −0.477, 0.634 (NS)
Dietary patterns	11.4316	10.6154	11.1000	*t*(158) = −1.124, 0.263 (NS)
Oral health status	1.6526	1.8000	1.7125	*t*(158) = 0.527, 0.599 (NS)

Note: NS = non-significant.

**Table 4:** Unstandardised (B) and standardised (β) regression coefficients and squared semi-partial correlations (sr^2^) for each predictor in a regression model predicting oral health status.

Variable	B [95% CI]	β	sr^2^
Food purchase	−0.083 [−0.308, 0.143]	−0.072	0.003364
Food preparation	−0.019 [−0.204, 0.165]	−0.022	0.000289
Food consumption	−0.041 [−0.213, 0.130]	−0.047	0.001444

## Data Availability

The data used to support the findings of this study are available from the corresponding author upon request.
